# *SCN11A* variants may influence postoperative pain sensitivity after gynecological surgery in Chinese Han female patients

**DOI:** 10.1097/MD.0000000000008149

**Published:** 2017-09-29

**Authors:** Jiaoli Sun, Guangyou Duan, Ningbo Li, Shanna Guo, Yuhao Zhang, Ying Ying, Mi Zhang, Qingli Wang, Jing Yu Liu, Xianwei Zhang

**Affiliations:** a Department of Anesthesiology, Tongji Hospital, Tongji Medical College, Huazhong University of Science and Technology, Wuhan; b Department of Anesthesiology, Xinqiao Hospital, Third Military Medical University, Chongqing; c Department of Anesthesiology, Rui Jin Hospital, Shanghai Jiao Tong University School of Medicine, Shanghai; d Department of Anesthesiology, Wuhan General Hospital of Guangzhou Military; e Key Laboratory of Molecular Biophysics of the Ministry of Education, College of Life Science and Technology and Center for Human Genome Research, Huazhong University of Science and Technology, Wuhan, China.

**Keywords:** gynecological surgery, postoperative pain sensitivity, *SCN11A*, single-nucleotide polymorphism

## Abstract

Nav1.9, encoded by sodium voltage-gated channel alpha subunit 11 (*SCN11A*), is one of the main sodium channels involved in pain transmission. Dysfunction of Nav1.9 alters pain sensitivity, resulting in insensitivity to pain or familial episodic pain. Our purpose was to explore the effects of *SCN11A* single-nucleotide polymorphisms (SNPs) on postoperative pain sensitivity in Chinese Han female patients after gynecological surgery.

Here, we combined the methods of tag SNPs and candidate SNPs. The associations between eleven *SCN11A* SNPs and basic pain sensitivity in female healthy volunteers were analyzed using the Plink software. The SNPs associated with basic pain sensitivity were termed positive *SCN11A* SNPs. The effect of these positive SNPs on postoperative pain sensitivity was explored in patients undergoing elective gynecological laparoscopic surgery and receiving postoperative patient-controlled analgesia (PCA). We assessed pain intensity using the numeric pain rating scale (NRS) and recorded PCA consumption.

Our results suggested that 5 SNPs (rs33985936, rs13080116, rs11720988, rs11709492, and rs11720013) in 11 tag and candidate SNPs were associated with basic pain sensitivity (*P* < .05). No evident association was found between the 5 positive SNPs and NRS (*P* > .05). However, among these positive SNPs, the minor alleles of rs33985936 and rs13080116 were significantly associated with increased PCA consumption (*P* < .01).

To our knowledge, this is the first study to report that *SCN11A* SNPs affect postoperative pain sensitivity in Chinese Han women after gynecological surgery. The SNP rs33985936 and rs13080116 may serve as novel predictors for postoperative pain.

## Abstract 2

Supplemental Digital Content is available in the text

## 1 Introduction

Sodium channels play an important role in pain transmission, and among the known sodium channels, Nav1.7, Nav1.8, and Nav1.9, which are predominantly expressed in peripheral nociceptive sensory neurons, have attracted much attention.^[1–3]^ Recently, the Liu group reported that sodium voltage-gated channel alpha subunit 11 (*SCN11A*) (encoding Nav1.9) mutations lead to the familial episodic pain.^[4]^ Meanwhile, previous research has showed that *SCN11A* dysfunction may result in a series of symptoms, such as congenital insensitivity to pain and familial episodic pain.^[4–6]^

Nav1.9 is predominantly expressed in small-diameter nociceptive sensory neurons, trigeminal ganglion neurons, and myenteric neurons,^[7,8]^ and plays a significant role in the maintenance of the resting potential polarization, therefore affects the excitability of neurons via the regulation of the resting potential.^[9–11]^

These findings indicate that Nav1.9 plays an important role in pain signal conduction and may influence pain sensitivity. Similar findings have been reported for *SCN9A* (encoding Nav1.7), whose mutations may lead to congenital insensitivity or extreme sensitivity to pain.^[12–15]^ Based on our previous findings that single-nucleotide polymorphisms (SNPs) in *SCN9A* (encoding Nav1.7) can affect basal and postoperative pain sensitivity,^[16–18]^ we speculated that there may also be an association between *SCN11A* and pain sensitivity.

Here, we first identified those SNPs that are highly associated with basal pain sensitivity in healthy volunteers (termed positive SNPs hereafter). Given that some factors (such as sex, age, environment, and disease) may affect pain sensitivity,^[19,20]^ the volunteers we recruited were all female college students with similar life styles and education experiences. We then explored the influence of these positive *SCN11A* SNPs on postoperative pain sensitivity in female patients who had undergone elective gynecological surgery (this patient population was selected to reduce confounding factors) and who then had access to postoperative patient-controlled analgesia (PCA).

## 2 Methods

### 2.1 Subjects

The study was approved by the ethical committee of Tongji Hospital, Huazhong University of Science and Technology, China, and registered on Clinical-Trials.gov (Identifier: NCT01950078). The volunteers and the patients were collected from August 2013 to August 2014, and written informed consents were obtained from the volunteers and the patients before the study.

**Figure 1:**
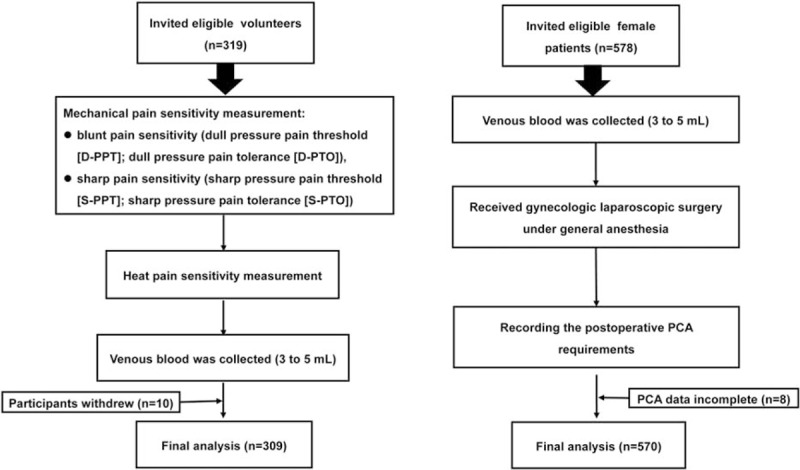
The flow diagram of the study.

As shown in Fig. 1, the healthy volunteers were used to identify the positive SNPs in *SCN11A* (SNPs associated with basal pain sensitivity). To reduce potential study bias stemming from variability in factors such as sex, age, environment, and underlying disease, we recruited 18 to 29-year-old Chinese Han female students with similar lifestyles and levels of education from Tongji Medical College of Huazhong University of Science and Technology. The inclusion criteria for volunteers were the absence of underlying diseases, chronic pain, and tobacco and alcohol abuse. Those who had taken painkillers within 1 month from the start of the study, had dermatitis, or were pregnant or lactating, and were excluded. Initially, 319 volunteers were included in the study. However, 10 dropped out of the study due to discomfort during blood collection, resulting in 309 volunteers in the study.

To explore the influence of positive *SCN11A* SNPs on postoperative pain sensitivity, we recruited 578 Chinese Han female patients, with American Society of Anesthesiologists statuses of I or II, and aged 18 to 65 years, who were scheduled for elective gynecologic laparoscopic surgery under general anesthesia. Patients with a history of chronic pain; severe cardiovascular diseases; diabetes mellitus; kidney or liver diseases; mental disorders; drug or alcohol addiction; communicating deficits; use of painkillers within 4 weeks before the start of the study; dermatitis, pregnancy, or lactation; or who refused participation in the study were excluded. In all, 570 cases were analyzed, as 8 patients were excluded due to incomplete PCA data.

### 2.2 Design

In this study, the healthy volunteers were used to identify the positive SNPs in *SCN11A* that were associated with basal pain sensitivity. Then, we explored the influence of the identified 5 positive *SCN11A* SNPs on postoperative pain sensitivity in the female patients undergoing elective gynecologic laparoscopic surgery.

In healthy volunteers, the basal pain sensitivity of all volunteers was detected through experimental pain measurement, including mechanical pain sensitivity and thermal pain sensitivity, as per standardized protocols. Five basal pain sensitivity-associated positive SNPs were found within *SCN11A* (rs33985936, rs13080116, rs11720988, rs11709492, and rs11720013), and the influence of these positive *SCN11A* SNPs on postoperative pain sensitivity was then investigated in the female patients. Postoperative pain intensity was assessed using the numeric pain rating scale (NRS), and PCA consumption was also recorded.

### 2.3 Mechanical pain sensitivity measurement

Mechanical pain sensitivity was measured. We measured blunt pain sensitivity (dull pressure pain threshold [D-PPT] and dull pressure pain tolerance [D-PTO]) and sharp pain sensitivity (sharp pressure pain threshold [S-PPT] and sharp pressure pain tolerance). The volunteers underwent 2 assessments each; the interval time between these 2 assessments was 10 minutes, and the average pain assessment values were recorded. The methods used have been previously described.^[16,21]^

### 2.4 Thermal pain sensitivity measurement

Thermal pain sensitivity was also measured according to the method of Montagne-Clave and Oliveras^[22]^ using an Ugo Basile-37370 thermal pain instrument made in Italian. The volunteers removed their fingers from the instrument as soon as they felt pain. Thermal pain sensitivity was analyzed through withdrawal lantency time. Their reaction times were recorded, and the average reaction times for the left and right hands were obtained.

### 2.5 Anesthetic technique

Patients were monitored after arrival in the operating room. Midazolam (0.05 mg/kg), propofol (2 mg/kg), sufentanil (0.5 μg/kg), and rocuronium (0.6 mg/kg) were administered to induce general anesthesia. Combined intravenous-inhalation anesthesia (remifentanil [0.2–0.4 μg/kg/min], propofol [6–10 mg/kg/h], and sevoflurane [1%–2%]) was administered to maintain of anesthesia, and muscle relaxants were prescribed as required.

### 2.6 Analgesia technique and assessment of postoperative pain

Postoperative analgesia was carried out by a specialized acute pain service (APS) team according to standard procedures. The day before the operation, the APS team visited the patients, conducted a simulation of PCA use, and instructed the patients in the use of the NRS. The patients were able to control analgesic consumption via a PCA pump according to their pain sensitivity. Parecoxib sodium (40 mg) was administered transvenously 15 minutes before starting the operation, and PCA (sufentanil [0.5 μg/mL] and tramadol [5 mg/mL]) was initiated as soon as the operation had been completed. NRS (at rest and moving) was recorded 30 minutes, 9 to 12 hours, and 21 to 24 hours after surgery. The maximum NRS during the postoperative follow-up period was used in the final analysis. The 24-hour postoperative PCA consumption was also recorded. Adverse effects were also recorded and interventional measures were carried out, as appropriate.

### 2.7 SNP selection and genotyping analysis

Eleven *SCN11A* SNPs were included in our study: the tag SNPs (rs13080116, rs11720988, rs4280575, rs4234134, rs12054380, rs11709492, rs11720013, and rs4637231) were selected based on phase 3 data from the HapMap Han Chinese in Beijing reference population database, and were identified using the Tagger program included in the Haploview v.4.2 software.^[23–25]^ In addition to these 8 SNPs, 2 additional SNPs (rs33985936 and rs72869687) were selected based on their position within the exons and the presence of amino acid substitutions (minor allele frequency >0.05). An additional SNP (rs4453791) was selected based on a previous study.^[26]^

Genomic DNA was extracted from venous blood obtained from participants, using the guanidinium isothiocyanate method, and then *SCN11A* SNPs were genotyped using ligase detection reactions carried out by the Shanghai BioWing Applied Biotechnology Company (http://www.biowing.com.cn/).

### 2.8 Structure prediction analysis of Nav1.9 DII to DIII due to Val909Ile

The structure is based on predictions obtained at http://zhanglab.ccmb.med.umich.edu/I-TASSER and is interpreted by pymol software.

### 2.9 Statistical analysis

The data of volunteers and patients were grouped according to the alleles of the *SCN11A* SNPs, and the effects of SNPs on basal and postoperative pain sensitivity were analyzed. All variables were described using standard descriptive statistics, such as the mean, SD, and frequency. The chi-square test was used to analyze the Hardy–Weinberg equilibrium (HWE) (*P* < .01 was excluded). For the volunteer samples, all genetic association analyses between *SCN11A* SNPs and pain sensitivity were conducted using Plinkv.1.07,^[27,28]^ with age and body mass index (BMI) as covariates. Three models (including additive, recessive, and dominant models) were considered. Analyses of association of SNPs within *SCN11A* and NRS or PCA requirement were performed using a linear regression analysis with age, tumor excision surgery (yes or no), and BMI as covariates. The additive model was considered. To avoid the potential impact of differences in patients’ weight on PCA consumption, the data of PCA requirement was analyzed as mL/kg of body weight. Analyses of variance were conducted using SPSS v.19.0 to detect differences in PCA requirements among patients carrying different *SCN11A* genotypes at rs33985936, and also differences in the NRS scores of patients carrying different *SCN11A* genotypes. A 2-tailed *P* value <.05 was considered to indicate statistical significance.

The online SHEsis software was used for linkage disequilibrium analysis.^[29]^

## 3 Results

### 3.1 SCN11A SNPs

All genotyped *SCN11A* SNPs in the volunteers are listed in Table 1. The total detection rate of *SCN11A* SNPs in healthy female volunteers was 0.989.

**Table 1:**
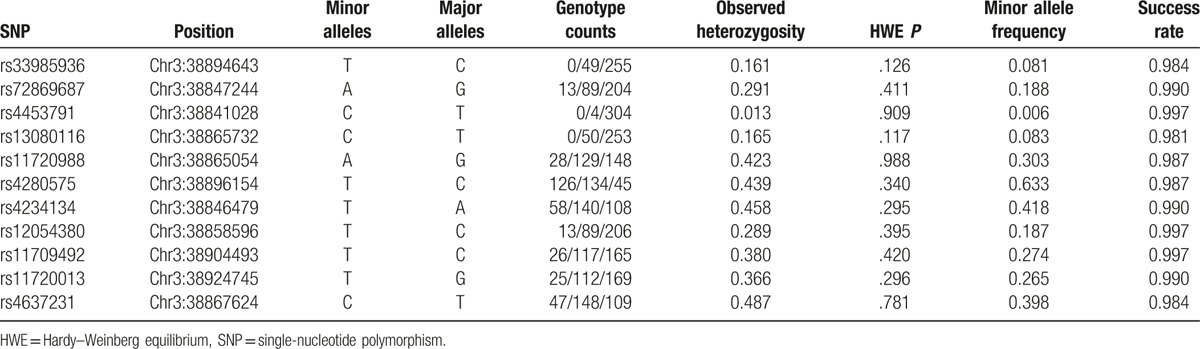
Genotyped *SCN11A* SNPs detected in volunteers.

### 3.2 Genetic association results between *SCN11A* SNPs and basal pain sensitivity

The 11 *SCN11A* SNPs all conformed to HWE testing and were used in the analysis. The minor allele was regarded as an acting gene. Therefore, β represented the effect and direction of the minor allele, negative numbers represented reduced pain threshold, and positive numbers represented increased pain threshold. Our results indicated that 5 SNPs within *SCN11A* were associated with basal pain sensitivity (Table 2) (statistical associations between 11 *SCN11A* SNPs and basal pain sensitivity are showed in Supplemental Content, Table S1). Of these 5 SNPs, the minor alleles of rs33985936 and rs13080116 were associated with D-PPT (*P* < .05), with β values of −0.351 and −0.345, respectively. This indicates that copies of the minor alleles (T/C) were associated with reductions in the D-PPT threshold by 0.351 and 0.345 kg/cm^2^, respectively. In addition, 3 SNPs (rs11720988, rs11709492, and rs11720013) were associated with S-PPT (*P* < .05). The β value was positive, indicating that the copies of the minor alleles in these 3 SNPs were associated with increases in S-PPT. In other words, each copy of the minor allele in rs11720988, rs11709492, and rs11720013 would increase the S-PPT threshold by an average of 0.655, 0.635, and 0.705 kg/cm^2^, respectively.

**Table 2:**
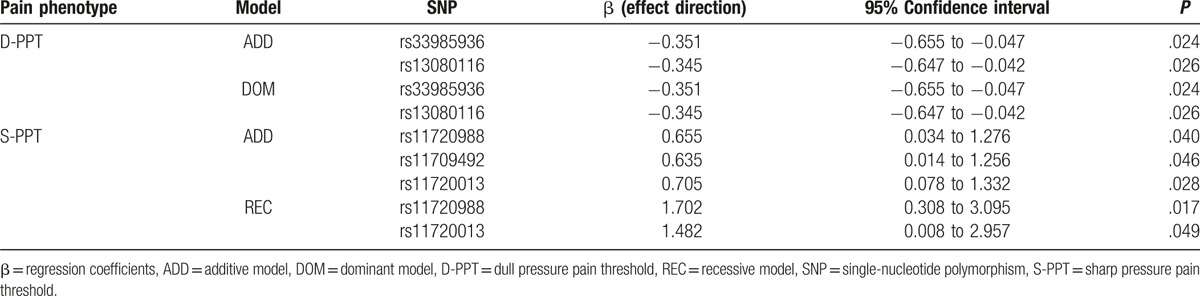
Statistical associations between the 5 positive *SCN11A* SNPs and basal pain sensitivity.

Linkage disequilibrium between rs33985936 and rs13080116 has been reported in the United States, Europe, and Australia.^[30]^ However, there have been no reports regarding these alleles for the Chinese Han population. Here we found that linkage disequilibrium between rs33985936 and rs13080116 also exists in the Chinese Han female population (*D*’ = 0.886 and *r*^2^ = 0.721 in the volunteers,).

### 3.3 Effects of positive *SCN11A* SNPs on NRS in patients

Our results indicate that there are no statistically significant associations between SNPs and NRS scores (*P* > .05) (Table 3).

**Table 3:**
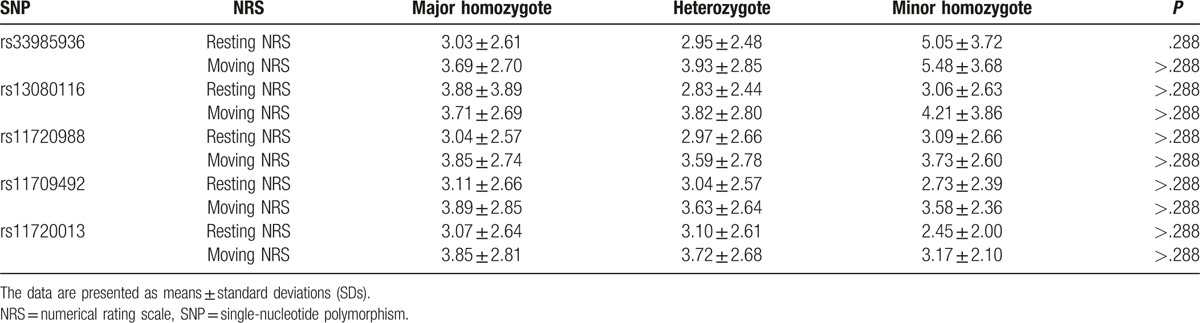
Maximum NRS in patients with different *SCN11A* SNPs.

### 3.4 Associations between the *SCN11A* SNPs and PCA consumption in patients

Linear regression analysis was used to explore the association between *SCN11A* SNPs and PCA consumption. Our results indicate that rs33895936 and rs13080116 were significantly associated with PCA consumption (*P* < .05; Table 4). We also found that there is linkage disequilibrium between rs33985936 and rs13080116 (*D*’ = 0.969, *r*^2^ = 0.760 in patients).

**Table 4:**
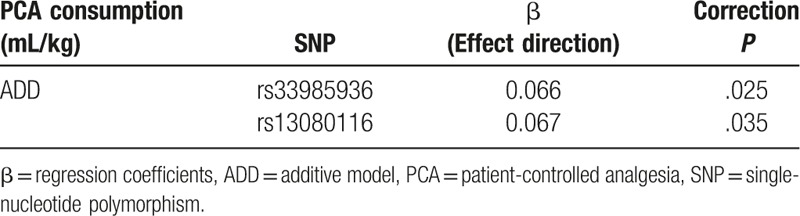
Statistical associations between *SCN11A* SNPs and PCA consumption in patients.

**Figure 2:**
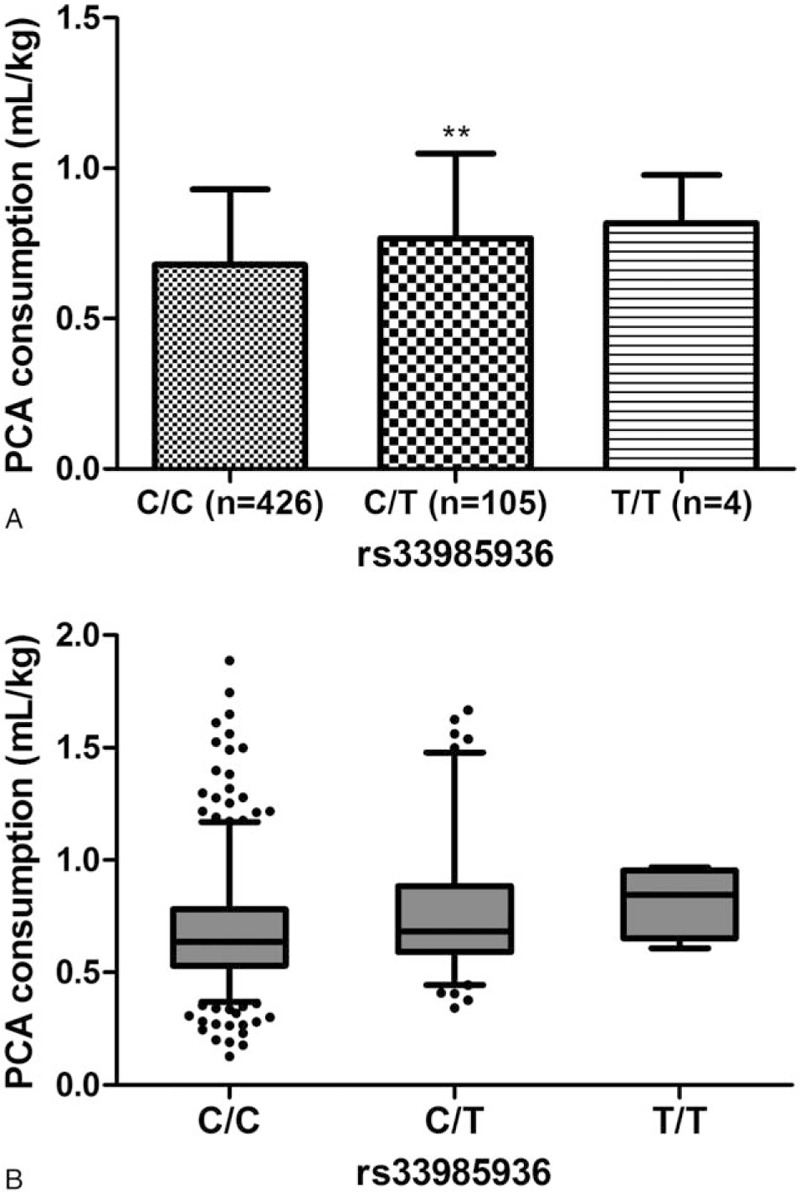
Patient-controlled analgesia (PCA) consumption in patients with different genotypes of rs33985936. (A, B) PCA consumption among rs33985936 C/C group, C/T group, and T/T group using histogram (^∗∗^*P* < .01 between C/C group and C/T) and plots, respectively.

Patient-controlled analgesia consumption was significantly different between patients with different genotypes of rs33985936. (T/T: 0.82 [SD 0.16] vs C/T: 0.77 [SD 0.28] vs C/C: 0.68 [SD 0.25]; *P* = .005; Fig. 2). Our results indicated that PCA consumption in the C/T group was significantly higher than in the C/C group (0.77 [SD: 0.28] vs 0.68 [SD: 0.25] mL/kg; *P* = .001; Fig. 2). In other words, PCA consumption in the C/T group was increased by about 13.2% compared with that in the C/C group.

### 3.5 The structure changes in Nav1.9 due to Val909Ile

**Figure 3:**
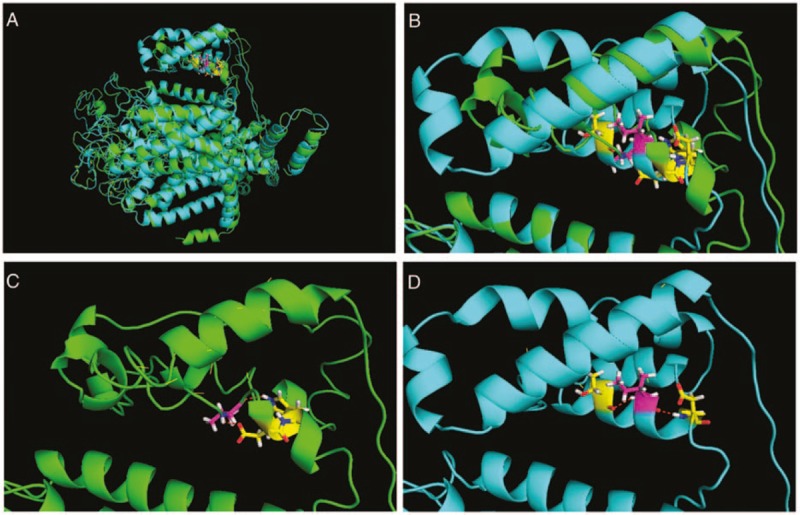
The structure model showing changes in Nav1.9 due to Val909Ile. (A) Nav1.9 DII to DIII. (B) A partial enlargement, focusing on the region near the residue 909 site. In the figures, overlapping wild-type (green) and variant-type (light blue) structures indicates that the structure has not changed, whereas regions without overlap indicate the structural changes. (C) Wild-type Val 909 has an irregular curl and forms hydrogen bonds with 912Asp and 913Trp. (D) Variant-type 909Ile leads to a change in an α-helix and forms hydrogen bonds with Thr906 and Asp912 (red dotted line represents a carbon atom, whereas the purple lines and yellow lines represent hydrogen bonds). The figure is based on predictions obtained on the web at http://zhanglab.ccmb.med.umich.edu/I-TASSER and interpreted using pymol software.

The amino acid substitution Val909Ile lies in the cytoplasmic loop between domains II and III of Nav1.9. Then, based on predictions obtained at http://zhanglab.ccmb.med.umich.edu/I-TASSER, we found that the Val909Ile results in the changes in intermolecular force and that this region becomes constricted structurally (Fig. 3).

## 4 Discussion

In this study, 5 SNPs (rs33985936, rs13080116, rs11720988, rs11709492, and rs11720013) detected within *SCN11A* were shown to be associated with the mechanical pain threshold (D-PPT and S-PPT) in healthy female volunteers. This suggests that these 5 positive *SCN11A* SNPs may be associated with basic pain sensitivity. Furthermore, we confirmed that, among these 5 positive *SCN11A* SNPs, rs33985936 and rs13080116 are significantly associated with PCA consumption in patients after gynecological laparoscopic surgery.

To reduce the potential effect of several factors, such as disease, demographics, and the environment, which may influence pain,^[20,31,32]^ we recruited healthy female college students, as they have a similar living environment and educational backgrounds, to identify positive SNPs. We also minimized potential bias by recruiting female patients undergoing the same type of surgery when assessing the association between these 5 positive SNPs and postoperative pain sensitivity.

The data obtained in the volunteers indicated that the 5 positive *SCN11A* SNPs were associated with basal pain sensitivity. Furthermore, the minor alleles of rs33985936 and rs13080116 (T and C, respectively) were associated with reduced D-PPT threshold. This indicated that subjects who carrying the minor allele of rs33985936 or rs13080116 might be more sensitive to pain. The minor alleles of rs11720988, rs11709492, and rs11720013 were associated with increased S-PPT threshold, indicating that subjects carrying minor alleles of these 3 SNPs might have lower pain sensitivity.

To the best of our knowledge, this is the first study to assess possible association between *SCN11A* SNPs and postoperative pain sensitivity. In this study, the patients were provided with adequate postoperative pain control through flexible PCA; therefore, the 5 positive *SCN11A* SNPs were not significantly associated with NRS score of patients. However, there were differences in PCA requirements in patients with different genotypes of rs33985936 and rs13080116. The minor allele in rs33985936 was associated with an increase of postoperative PCA consumption of about 13.2%. As PCA opioid consumption represents the actual demand for relief of postoperative pain in surgical patients, and thus can be considered to reflect the postoperative pain in patients,^[33]^ we speculated that rs33985936 and rs13080116 might affect postoperative pain sensitivity in female patients after gynecological laparoscopic surgery. We found that the minor alleles of these *SCN11A* SNPs were indeed associated with increased postoperative pain sensitivity. We also observed that the minor allele of rs33985936 was associated with changes in pain sensitivity in patients, in the same as the direction as the changes in bias basic pain sensitivity observed in the volunteers.

Although linkage disequilibrium between rs33985936 and rs13080116 has been reported in the United States, Europe, and Australia,^[30]^ there have been no reports regarding these SNPs in the Chinese Han population. We demonstrated linkage disequilibrium between rs33985936 and rs13080116 in the Chinese Han female population (*D*’ = 0.886 and *r*^2^ = 0.721 in volunteers; and *D*’ = 0.969, *r*^2^ = 0.760 in patients). Because rs13080116 is located in an intronic area, whereas rs33985936 is located in an exon and induces an amino acid substitution (Val909Ile), we will only consider the rs33985936 SNP in the following discussion.

**Figure 4:**
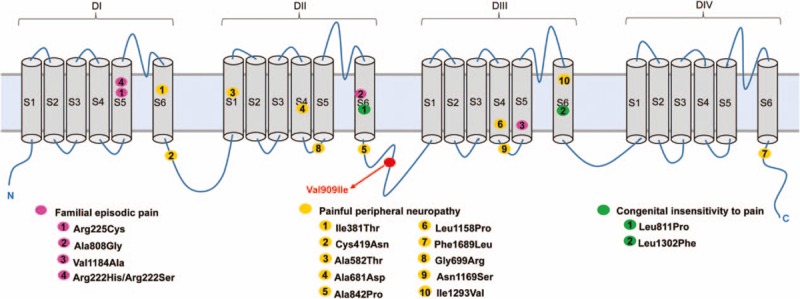
Variants of Nav1.9 that are associated with pain disorders: familial episodic pain, painful peripheral neuropathy, and congenital insensitivity to pain. A schematic of the sodium channel Nav1.9 α-subunit, which has 4 domains, each of which consists of 6 transmembrane segments. The locations of the currently known Nav1.9 variants that are associated with pain disorders are shown.

Voltage-gated sodium channel Nav1.9, encoded by *SCN11A*, is highly expressed in peripheral nociceptive neurons and is considered to be a key regulator of nociceptor excitability.^[9,34]^ Recent studies in humans have indicated that Nav1.9 dysfunction caused by certain *SCN11A* variants associated with a series of pain disorders (Fig. 4). Previous studies have shown that gain-of-function mutations in *SCN11A* are been linked to painful peripheral neuropathy^[5,35,36]^ and familial episodic pain.^[4,37,38]^ In fact, Zhang et al^[4]^ have identified gain-of-function mutations in *SCN11A* (Arg225Cys and Ala808Gly) that increased electrical activity and promoted action potential firing in dorsal root ganglion neurons in 2 Chinese families with episodic pain. On the contrary, other studies have found that gain-of-function mutations (Leu811Pro and Leu1302Phe) in *SCN11A* lead to an inability to feel pain.^[6,39]^ These studies have demonstrated a role for Nav1.9 in human pain. However, in contrast to the rare variants that cause the pain disorders, other studies have suggested that more common SNPs in some genes can lead to quantitative rather than qualitative changes in pain sensitivity.^[40]^ Therefore, we hypothesized that *SCN11A* SNPs may also be involved in the regulation of pain sensitivity.

In this study, we identified the *SCN11A* SNP rs33985936 (2725C > T), which leads to the amino acid substitution Val909Ile, as being associated with postoperative pain. This variant was first reported in a Japanese family with childhood episodic pain syndrome, wherein the affected patients carried the Arg222His and Val909Ile mutations.^[38]^ However, functional analysis was not performed previously. The amino acid residue Val909 lies in the cytoplasmic loop between domain II and III of Nav1.9, and Val909Ile results in the changes in intermolecular force; in addition, this region becomes more closely structurally (Fig. 3), which might affect the function of sodium channel Nav1.9. Furthermore, although the role of the cytoplasmic loop between domains II and III of the sodium channel is not clear, the dysfunctions in this region of Nav1.7 and Nav1.8, 2 other important sodium channels regulating pain, have been linked to human pain sensitivity. Specifically, Ala1073Val in Nav1.8 is associated with biased human pain sensitivity,^[41]^ and gain-of-function changes in this region of Nav1.7 have been associated with painful diseases, such as inherited erythromelalgia (Del-Leu955, Arg1150Trp),^[42,43]^ paroxysmal extreme pain disorder (Arg996Cys and Val1298Asp),^[44]^ and small fiber neuropathy (Met932Leu and Val991Ile).^[45]^ Based on these studies, we speculate that the amino acid substitution Val909Ile in Nav1.9 may increase postoperative pain sensitivity, potentially by increasing the excitability of nociceptive neurons resulting from structural and functional changes in the loop between domains II and III in Nav1.9.

Our study has some limitations. First, to reduce the false-negative rate, we did not perform multiple tests to adjust the *P* value in the volunteers. Second, while rs33985936 (2725C >T) leads to the amino acids change Val909Ile, the influence of rs33985936 on the electrophysiology of sodium channel Nav1.9 is yet unclear. Further research is required to investigate this relationship to better explain the exact mechanism of rs33985936 T allele increasing pain sensitivity.

## 5 Conclusions

*SCN11A* SNPs are associated with pain sensitivity. More specifically, the minor alleles of rs33985936 and rs13080116 are associated with increased postoperative pain sensitivity in patients after gynecological surgery. The SNPs rs33985936 and rs13080116 may serve as novel predictors for postoperative pain.

## Supplementary Material

Supplemental Digital Content
