# Supplementary material for: SCN11A variants may influence postoperative pain sensitivity after gynecological surgery in Chinese Han female patients
Source: Medicine (Baltimore). 2017 Sep 29;96(39):e8149. doi: 10.1097/MD.0000000000008149 (PMC5626299; doi:10.1097/MD.0000000000008149)
Supplement: Supplemental Digital Content [file medi-96-e8149-s001.docx]

**Supplemental Content**

# *SCN11A* variants may influence postoperative pain sensitivity after gynecological surgery in Chinese Han female patients

Jiaoli Sun, Guangyou Duan, Ningbo Li, Shanna Guo, Yuhao Zhang, Ying Ying, Mi Zhang, Qingli Wang Jing Yu Liu, Xianwei Zhang

**Supplemental Content**

**Table S1 Statistical associations between eleven *SCN11A* SNPs and basal pain sensitivity**

| **Pain phenotype** | **Model** | **SNP** | **β (effect direction)** | **95%**  **Confidence interval** | ***P*-value** |
| --- | --- | --- | --- | --- | --- |
| **D-PPT** | ADD | rs33985936 | -0.351 | -0.655 – -0.047 | .024 |
|  |  | rs13080116 | -0.345 | -0.647 – -0.042 | .026 |
|  |  | rs4453791 | -0.201 | -1.188 – -0.786 | .690 |
|  |  | rs4234134 | 0.089 | -0.067 – -0.245 | .266 |
|  |  | rs72869687 | -0.051 | -0.251 – 0.149 | .619 |
|  |  | rs12054380 | -0.040 | -0.240 – 0.159 | .692 |
|  |  | rs11720988 | 0.022 | -0.152 – 0.195 | .807 |
|  |  | rs4637231 | -0.073 | -0.237 – 0.091 | .382 |
|  |  | rs4280575 | -0.067 | -0.227 – 0.094 | .416 |
|  |  | rs11709492 | 0.021 | -0.152 – 0.194 | .812 |
|  |  | rs11720013 | 0.033 | -0.142 – 0.207 | .715 |
|  | DOM | rs33985936 | -0.351 | -0.655 – -0.047 | .024 |
|  |  | rs13080116 | -0.345 | -0.647 – -0.042 | .026 |
|  |  | rs4453791 | -0.201 | -1.188 – 0.786 | .690 |
|  |  | rs4234134 | 0.097 | -0.139 – 0.332 | .421 |
|  |  | rs72869687 | -0.025 | -0.265 – 0.216 | .840 |
|  |  | rs12054380 | -0.011 | -0.252 – 0.229 | .927 |
|  |  | rs11720988 | 0.075 | -0.150 – 0.301 | .513 |
|  |  | rs4637231 | -0.073 | -0.308 – 0.163 | .546 |
|  |  | rs4280575 | -0.046 | -0.274 – 0.182 | .692 |
|  |  | rs11709492 | 0.077 | -0.147 – 0.301 | .499 |
|  |  | rs11720013 | 0.097 | -0.129 – 0.322 | .402 |
|  | REC | rs4453791 | – | – | – |
|  |  | rs4234134 | 0.155 | -0.131 – 0.441 | .290 |
|  |  | rs72869687 | -0.260 | -0.814 – 0.295 | .360 |
|  |  | rs12054380 | -0.253 | -0.808 – 0.302 | .373 |
|  |  | rs11720988 | -0.115 | -0.505 – 0.274 | .562 |
|  |  | rs13080116 | – | – | – |
|  |  | rs4637231 | -0.137 | -0.449 – 0.175 | .389 |
|  |  | rs33985636 | – | – | – |
|  |  | rs4280575 | -0.172 | -0.489 – 0.146 | .290 |
|  |  | rs11709492 | -0.136 | -0.538 – 0.266 | .508 |
|  |  | rs11720013 | -0.139 | -0.549 – 0.271 | .507 |
| **D-PTO** | ADD | rs4453791 | -0.425 | -2.169 – 1.32 | .634 |
|  |  | rs4234134 | -0.042 | -0.320 – 0.235 | .764 |
|  |  | rs72869687 | 0.085 | -0.269 – 0.439 | .638 |
|  |  | rs12054380 | 0.096 | -0.257 – 0.449 | .594 |
|  |  | rs11720988 | 0.065 | -0.240 – 0.371 | .676 |
|  |  | rs13080116 | -0.247 | -0.783 – 0.290 | .368 |
|  |  | rs4637231 | -0.033 | -0.323 – 0.257 | .824 |
|  |  | rs33985636 | -0.393 | -0.932 – 0.146 | .154 |
|  |  | rs4280575 | -0.062 | -0.345 – 0.221 | .668 |
|  |  | rs11709492 | 0.101 | -0.204 – 0.407 | .516 |
|  |  | rs11720013 | 0.052 | -0.257 – 0.361 | .741 |
|  | DOM | rs4453791 | -0.425 | -2.169 – 1.32 | .634 |
|  |  | rs4234134 | -0.017 | -0.434 – 0.400 | .936 |
|  |  | rs72869687 | 0.229 | -0.196 – 0.654 | .292 |
|  |  | rs12054380 | 0.244 | -0.181 – 0.668 | .261 |
|  |  | rs11720988 | 0.161 | -0.237 – 0.558 | .429 |
|  |  | rs13080116 | -0.247 | -0.783 – 0.290 | .368 |
|  |  | rs4637231 | -0.022 | -0.438 – 0.394 | .917 |
|  |  | rs33985636 | -0.393 | -0.932 – 0.146 | .154 |
|  |  | rs4280575 | -0.049 | -0.451 – 0.353 | .811 |
|  |  | rs11709492 | 0.280 | -0.115 – 0.675 | .165 |
|  |  | rs11720013 | 0.197 | -0.202 – 0.595 | .335 |
|  | REC | rs4453791 | – | – | – |
|  |  | rs4234134 | -0.117 | -0.624 – 0.390 | .652 |
|  |  | rs72869687 | -0.564 | -1.544 – 0.417 | .261 |
|  |  | rs12054380 | -0.557 | -1.538 – 0.423 | .266 |
|  |  | rs11720988 | -0.149 | -0.836 – 0.538 | .671 |
|  |  | rs13080116 | – | – | – |
|  |  | rs4637231 | -0.080 | -0.632 – 0.472 | .776 |
|  |  | rs33985636 | – | – | – |
|  |  | rs4280575 | -0.148 | -0.707 – 0.412 | .605 |
|  |  | rs11709492 | -0.356 | -1.066 – 0.354 | .327 |
|  |  | rs11720013 | -0.361 | -1.086 – 0.364 | .330 |
| **S-PPT** | ADD | rs11720988 | 0.655 | 0.034 – 1.276 | .040 |
|  |  | rs11709492 | 0.635 | 0.014 – 1.256 | .046 |
|  |  | rs11720013 | 0.705 | 0.078 – 1.332 | .028 |
|  |  | rs33985936 | -1.085 | -2.186 – 0.016 | .054 |
|  |  | rs13080116 | -0.958 | -2.052 – 0.136 | .087 |
|  |  | rs4453791 | -3.072 | -6.621 – 0.478 | .091 |
|  |  | rs4234134 | -0.118 | -0.683 – 0.447 | .682 |
|  |  | rs72869687 | -0.251 | -0.972 – 0.470 | .496 |
|  |  | rs12054380 | -0.214 | -0.935 – 0.508 | .562 |
|  |  | rs4637231 | 0.304 | -0.285 – 0.893 | .313 |
|  |  | rs4280575 | 0.262 | -0.317 – 0.841 | .376 |
|  | REC | rs11720988 | 1.702 | 0.308 – 3.095 | .017 |
|  |  | rs11720013 | 1.482 | 0.008 – 2.957 | .049 |
|  |  | rs33985936 | – | – | – |
|  |  | rs13080116 | – | – | – |
|  |  | rs4453791 | – | – | – |
|  |  | rs4234134 | 0.120 | -0.914 – 1.154 | .821 |
|  |  | rs72869687 | 0.349 | -1.654 – 2.352 | .733 |
|  |  | rs12054380 | 0.370 | -1.637 – 2.378 | .718 |
|  |  | rs4637231 | 1.033 | -0.082 – 2.148 | .071 |
|  |  | rs4280575 | 1.043 | -0.098 – 2.184 | .074 |
|  |  | rs11709492 | 1.387 | -0.059 – 2.832 | .061 |
|  | DOM | rs4453791 | -3.072 | -6.621 – 0.478 | .091 |
|  |  | rs4234134 | -0.349 | -1.198 – 0.501 | .422 |
|  |  | rs72869687 | -0.429 | -1.296 – 0.438 | .333 |
|  |  | rs12054380 | -0.379 | -1.246 – 0.489 | .393 |
|  |  | rs11720988 | 0.541 | -0.272 – 1.353 | .193 |
|  |  | rs13080116 | -0.958 | -2.052 – 0.136 | .087 |
|  |  | rs4637231 | 0.037 | -0.809 – 0.883 | .932 |
|  |  | rs33985636 | -1.085 | -2.186 – 0.016 | .054 |
|  |  | rs4280575 | -0.010 | -0.834 – 0.814 | .981 |
|  |  | rs11709492 | 0.634 | -0.172 – 1.44 | .124 |
|  |  | rs11720013 | 0.728 | -0.084 – 1.541 | .080 |
| **S-PTO** | ADD | rs4453791 | -2.292 | -11.59 – 7.005 | .629 |
|  |  | rs4234134 | 0.252 | -1.226 – 1.73 | .739 |
|  |  | rs72869687 | 0.368 | -1.519 – 2.254 | .703 |
|  |  | rs12054380 | 0.439 | -1.443 – 2.321 | .648 |
|  |  | rs11720988 | 0.129 | -1.499 – 1.758 | .876 |
|  |  | rs13080116 | -1.45 | -4.303 – 1.403 | .32 |
|  |  | rs4637231 | -0.560 | -2.106 – 0.987 | .479 |
|  |  | rs33985636 | -2.009 | -4.886 – 0.867 | .172 |
|  |  | rs4280575 | -0.319 | -1.828 – 1.19 | .679 |
|  |  | rs11709492 | 0.337 | -1.293 – 1.968 | .685 |
|  |  | rs11720013 | 0.346 | -1.302 – 1.994 | .681 |
|  | DOM | rs4453791 | -2.292 | -11.59 – 7.005 | .629 |
|  |  | rs4234134 | 0.920 | -1.302 – 3.142 | .418 |
|  |  | rs72869687 | 0.486 | -1.784 – 2.757 | .675 |
|  |  | rs12054380 | 0.581 | -1.683 – 2.845 | .616 |
|  |  | rs11720988 | 0.005 | -2.116 – 2.127 | .996 |
|  |  | rs13080116 | -1.45 | -4.303 – 1.403 | .32 |
|  |  | rs4637231 | -0.610 | -2.828 – 1.609 | .591 |
|  |  | rs33985636 | -2.009 | -4.886 – 0.867 | .172 |
|  |  | rs4280575 | -0.428 | -2.572 – 1.717 | .696 |
|  |  | rs11709492 | 0.634 | -1.476 – 2.743 | .556 |
|  |  | rs11720013 | 0.534 | -1.595 – 2.663 | .623 |
|  | REC | rs4453791 | – | – | – |
|  |  | rs4234134 | -0.517 | -3.22 – 2.187 | .708 |
|  |  | rs72869687 | 0.246 | -4.993 – 5.485 | .927 |
|  |  | rs12054380 | 0.292 | -4.944 – 5.528 | .913 |
|  |  | rs11720988 | 0.639 | -3.024 – 4.301 | .733 |
|  |  | rs13080116 | – | – | – |
|  |  | rs4637231 | -0.951 | -3.893 – 1.99 | 0.527 |
|  |  | rs33985636 | – | – | – |
|  |  | rs4280575 | -0.420 | -3.406 – 2.565 | .783 |
|  |  | rs11709492 | -0.222 | -4.013 – 3.57 | .909 |
|  |  | rs11720013 | 0.143 | -3.727 – 4.014 | .942 |
| **WLT** | ADD | rs4453791 | -2.237 | -5.462 – 0.987 | .175 |
|  |  | rs4234134 | -0.417 | -0.927 – 0.094 | .111 |
|  |  | rs72869687 | 0.567 | -0.084 – 1.218 | .089 |
|  |  | rs12054380 | 0.565 | -0.087 – 1.216 | .090 |
|  |  | rs11720988 | 0.045 | -0.518 – 0.608 | .876 |
|  |  | rs13080116 | -0.042 | -1.032 – 0.947 | .933 |
|  |  | rs4637231 | 0.102 | -0.434 – 0.638 | .708 |
|  |  | rs33985636 | 0.241 | -0.756 – 1.238 | .636 |
|  |  | rs4280575 | 0.227 | -0.295 – 0.748 | .395 |
|  |  | rs11709492 | 0.245 | -0.322 – 0.811 | .398 |
|  |  | rs11720013 | 0.213 | -0.358 – 0.783 | .465 |
|  | DOM | rs4453791 | -2.237 | -5.462 – 0.987 | .175 |
|  |  | rs4234134 | -0.343 | -1.114 – 0.427 | .383 |
|  |  | rs72869687 | 0.493 | -0.293 – 1.278 | .220 |
|  |  | rs12054380 | 0.491 | -0.295 – 1.277 | .222 |
|  |  | rs11720988 | 0.123 | -0.61 – 0.857 | .742 |
|  |  | rs13080116 | -0.042 | -1.032 – 0.947 | .933 |
|  |  | rs4637231 | 0.073 | -0.696 – 0.842 | .853 |
|  |  | rs33985636 | 0.241 | 0.756 – 1.238 | .636 |
|  |  | rs4280575 | 0.359 | -0.382 – 1.099 | .344 |
|  |  | rs11709492 | 0.440 | -0.292 – 1.172 | .240 |
|  |  | rs11720013 | 0.528 | -0.207 – 1.264 | .160 |
|  | REC | rs4453791 | – | – | – |
|  |  | rs4234134 | -0.886 | -1.818 – 0.046 | .064 |
|  |  | rs72869687 | 1.752 | -0.054 – 3.558 | .058 |
|  |  | rs12054380 | 1.744 | -0.066 – 3.554 | .060 |
|  |  | rs11720988 | -0.141 | -1.407 – 1.126 | .828 |
|  |  | rs13080116 | – | – | – |
|  |  | rs4637231 | 0.242 | -0.777 – 1.261 | .642 |
|  |  | rs33985636 | – | – | – |
|  |  | rs4280575 | 0.192 | -0.840 – 1.224 | .716 |
|  |  | rs11709492 | 0.098 | -1.416 – 1.221 | .885 |
|  |  | rs11720013 | -0.572 | -1.911 – 0.767 | .403 |

**Abbreviation:** SNP, single nucleotide polymorphism; ADD, additive model; DOM, dominant model; REC, recessive model; β, regression coefficients; D-PPT, dull pressure pain threshold; D-PTO, dull pressure pain tolerance S-PPT, sharp pressure pain threshold, S-PTO=sharp pressure pain tolerance, WLT=withdrawal lantency time.
